# Common patient-reported outcomes across ICHOM Standard Sets: the potential contribution of PROMIS®

**DOI:** 10.1186/s12911-021-01624-5

**Published:** 2021-09-06

**Authors:** Caroline B. Terwee, Marloes Zuidgeest, Harald E. Vonkeman, David Cella, Lotte Haverman, Leo D. Roorda

**Affiliations:** 1 Department of Epidemiology and Data Science, Amsterdam Public Health Research Institute, Amsterdam UMC, Vrije Universiteit Amsterdam, De Boelelaan 1089a, 1081 HV Amsterdam, The Netherlands; 2 National Health Care Institute, Diemen, The Netherlands; 3 Department of Rheumatology and Clinical Immunology, Medisch Spectrum Twente, Enschede, The Netherlands; 4 Department of Psychology, Health and Technology, University of Twente, Enschede, The Netherlands; 5 Department of Medical Social Sciences, Northwestern University Feinberg School of Medicine, Chicago, USA; 6 Psychosocial Department, Emma Children’s Hospital Amsterdam UMC, University of Amsterdam, Meibergdreef 9, Amsterdam, The Netherlands; 7 Amsterdam Rehabilitation Research Center | Reade, Amsterdam, The Netherlands

**Keywords:** Patient-reported outcomes, Outcome measurement, Healthcare evaluation

## Abstract

**Background:**

The International Consortium for Health Outcomes Measurement (ICHOM) develops condition-specific Standard Sets of outcomes to be measured in clinical practice for value-based healthcare evaluation. Standard Sets are developed by different working groups, which is inefficient and may lead to inconsistencies in selected PROs and PROMs. We aimed to identify common PROs across ICHOM Standard Sets and examined to what extend these PROs can be measured with a generic set of PROMs: the Patient-Reported Outcomes Measurement Information System (PROMIS®).

**Methods:**

We extracted all PROs and recommended PROMs from 39 ICHOM Standard Sets. Similar PROs were categorized into unique PRO concepts. We examined which of these PRO concepts can be measured with PROMIS.

**Results:**

A total of 307 PROs were identified in 39 ICHOM Standard Sets and 114 unique PROMs are recommended for measuring these PROs. The 307 PROs could be categorized into 22 unique PRO concepts. More than half (17/22) of these PRO concepts (covering about 75% of the PROs and 75% of the PROMs) can be measured with a PROMIS measure.

**Conclusion:**

Considerable overlap was found in PROs across ICHOM Standard Sets, and large differences in terminology used and PROMs recommended, even for the same PROs. We recommend a more universal and standardized approach to the selection of PROs and PROMs. Such an approach, focusing on a set of core PROs for all patients, measured with a system like PROMIS, may provide more opportunities for patient-centered care and facilitate the uptake of Standard Sets in clinical practice.

## Background

Measuring outcomes that matter to patients—including Patient-Reported Outcomes (PROs, Table 1)—can help healthcare providers to benchmark treatment effects against their peers and, by identifying and learning from best practices, provide opportunities to improve quality of care [1–3].

**Table 1:** Glossary of terms

Abbreviation	Full text	Definition	Examples
PRO	Patient-reported outcome	Any report of the status of a patient’s health condition that comes directly from the patient, without interpretation of the patient’s response by a clinician or anyone else [61]	Depression Pain
PROM	Patient-reported outcome measure	A questionnaire used to measure the PRO	Patient Health Questionnaire (PHQ-9) Visual Analogue Scale (VAS) for pain
PROMIS	Patient-reported outcomes measurement information system	A set of generic PROMs that can be used as short forms or CAT to measure aspects of physical, mental, and social health in adults and children across medical conditions	www.healthmeasures.net/promis
PROMIS measure	Patient-reported outcomes measurement information system measure	A PROM (short form or CAT) from the PROMIS system	PROMIS® Item Bank V1.0 Depression-Short Form 8a PROMIS® Item Bank V1.1 Pain Interference CAT

*CAT* Computerized Adaptive Test, where a computer algorithm consecutively selects questions from a large set of questions (item bank), based on responses to previous questions

In 2012, the International Consortium for Health Outcomes Measurement (ICHOM) was founded with the aim to define Standard Sets of outcomes for every major medical condition [4]. Standard Sets are minimum sets of outcomes that matter most to patients. These sets should be measured and reported in all patients with a specific condition (or disease). Standard Sets have been developed using a standardized consensus methodology among a team of experts and patient representatives in the field [5]. Up to May 2021, 39 Standard Sets were published and another five were in progress [4, 6–35].

A potential barrier for implementing ICHOM Standard Sets is that they are independently developed by different working groups. Although it is important that Standard Sets are developed by people who have expertise in the particular condition, collaboration and harmonization across Standard Sets is currently limited, which leads to large differences and inconsistencies in selected PROs, terminology used, and recommended Patient-Reported Outcome Measures (PROMs), even for the same PROs [4, 6–17]. This complicates the implementation and use of Standard Sets in clinical practice.

A system of common data elements across conditions could improve the situation and may speed up the development and uptake of Standard Sets considerably. Generally, all people want to feel and function ‘normally’, i.e., live without symptoms, such as pain, fatigue or depression, and be able to carry out daily activities and social roles. These feelings and functions can be affected by different health conditions. For example, climbing stairs can be affected by knee osteoarthritis (e.g. because of pain), lung disease (e.g. because of breathlessness) of heart failure (e.g. because of fatigue). Different conditions can result in the same patient-reported problem; in this case, difficulty climbing stairs. Because human values, experiences, and desires cut across health status, there is considerable overlap in relevant PROs across conditions [36].

Ideally, these non-condition specific (or common) outcomes could be measured with just one set of universal (i.e. generic) PROMs across conditions. This is, however, currently not the case. A large number of different PROMs is being used for measuring common outcomes within and between different patient groups. One reason for this is that it has been considered important to develop disease-specific PROMs, while for common outcomes like pain or fatigue, this may not be necessary. Another reason is that many PROMs were developed because of criticism on the content or insufficient measurement properties of existing PROMs. However, with recent methodological innovations in PROM development, such as application of item response theory (IRT), universal PROMs have been developed with good measurement properties, that can be applied across medical conditions, including patients without a medical diagnosis and patients with multiple (chronic) conditions [37, 38]. One such cross-cutting IRT-based measurement option is the Patient-Reported Outcomes Measurement Information System (PROMIS®, Table 1). PROMIS researchers developed a conceptual framework of commonly relevant PROs across the broad domains of physical, mental, and social functioning. They also developed PROMs within each of these domains, that are universally applicable across patients populations [39, 40]. PROMIS researchers used IRT to create item banks (i.e. large sets of questions), enabling the possibility of applying short forms where needed and computerized adaptive tests (CAT) where possible, that yield highly reliable and comparable scores with a few relevant items only [41–43].

The aim of this study was to identify common PROs across ICHOM Standard Sets and examine the extent to which these PROs can be measured with PROMIS.

## Methods

One author (MZ) downloaded the reference guides of all 39 available ICHOM Standard Sets from the ICHOM website on June 2021 and extracted all individuals PROs and recommended PROMs (including single items, unidimensional scales, and multidimensional questionnaires) [4]. The literal PRO terminology used in the reference guides was extracted. Another author (CT) checked the data extraction, and classified all PROs within the domains of global health, physical health, mental health, and social health, in accordance with the PROMIS conceptual framework. A third author (DC) reviewed the classification. Finally, one author (CT) searched the HealthMeasures website [44] to examine which of these PROs can be measured with a PROMIS measure.

## Results

A total of 307 PROs were extracted from the 39 Standard Sets (Table 2). The number of PROs included in these Standard Sets varies from 1 (cataract) to 27 (Parkinson’s disease). The 307 PROs refer to 22 unique PRO concepts, three in the domain of global health (general health status, health-related quality of life, quality of life), fourteen in the domain of physical health (six included in the PROMIS profile (i.e. core) domains: fatigue, pain intensity, physical function, mobility, upper extremity function, sleep disturbances; three included in the PROMIS additional Domains: dyspnea, gastrointestinal symptoms, sexual function; and five that are not included in the PROMIS conceptual framework: hearing, speech/communication, urinary symptoms, vision, other), four in the domain of mental health (general mental health, anxiety, depression, cognitive function), and one in the domain of social health (ability to participate in social roles/peer relationships) (Table 2). The most commonly included PROs are ability to participate in social roles/peer relationships (included in 25 out of 39 Standard Sets, of which 11 used the same term, i.e. ‘social function(ing)’), physical function (included in 21 out of 39 Standard Sets, of which 10 used the same term, i.e. ‘physical function(ing)’), health-related quality of life (included in 18 Standard Sets), pain intensity (included in 17 Standard Sets, of which 12 used the same term, i.e. ‘pain’), depression (included in 16 Standard Sets), general mental health (included in 14 Standard Sets), anxiety (included in 12 Standard Sets), and fatigue and overall quality of life (both included in 10 Standard Sets)..

**Table 2:** Patient-Reported Outcomes included in ICHOM Standard Sets, classified according to the PROMIS domain framework [62]

	PROMIS Global Health domain			PROMIS profile domains										PROMIS additional domains				Non-PROMIS domains					Total
				Physical health						Mental health			Social health	Physical health			Mental health	Physical health					
				Physical function																			
	General health status	Health-related quality of life	Quality of life	Fatigue	Pain	General	Mobility	Upper extre-mity	Sleep distur-bance	General	Anxiety	Depres-sion	Ability to participate in social roles/peer relation-ships	Dyspnea	Gastro-intestinal Symptoms	Sexual function	Cognitive function	Hearing	Speech communication	Urinary symp-toms	Vision	Other	
Addiction	1					1				1			1									5	10
Adult oral health					1				1				2						1			4	9
Advanced prostate cancer				1	1	1				1					1	1				1		1	8
Artrial fibrilation		1				1				1			1				1					1	6
Breast cancer			1	1	1	1		1		1	1	1	2			2	1					5	18
Cataract																					1		1
Chronic kidney disease		1		1	1	2						1											6
Cleft lip and palate											1	1	2	1					1			5	11
Colorectal cancer			1	1	1	1	1			1		1	1		6	3						1	18
Congetinal heart disease		1	1								1	1	1										5
Congenital upper limb anomalies		1						1		1												1	4
Coronary artery disease		1				1						1		1								1	5
COVID-19		1								1	1	1	1	1	1		1				1	1	10
Craniofacial microsomia		1				1					1	1	1	1				1	1		1	4	13
Dementia			1		1																	1	3
Depression and anxiety						1					4	1	2									3	11
Depression and anxiety for children and young people	2										1	2										2	7
Diabetes			1									1										1	3
Hand and wrist conditions		1			1	1		1					1									1	6
Heart failure		1		1		2			1		1	1	1									2	10
Hip and knee osteoarthritis			1		1	1							1										4
Hypertension in low and middle income countries			1													1							2
Inflammatory arthritis	1			1	1	1							1										5
Inflammatory bowel disease				1	1						1				2							3	8
Localized prostate cancer															1	1				2		1	5
Low back pain		1			2	1							1										5
Lung cancer	1		1	1	1	1				1			1	2			1						10
Macula degeneration							1			1											1		4
Older person					1	1				1			2					1					6
Overactive bladder		1														1						1	3
Overall adult health	2			2	1	1	1		1	1	1	1	3					1			1		16
Overall pediatric health	1	1			1		1		1	1			1			1	1	1	1		1	1	13
Parkinson's disease		1	1	1	1	5	1		2				2		2	1	1		1	1		7	27
Pediatric facial palsy		1											1									2	4
Personality disorders		1				2				3	1		2										9
Pregnancy and childbirth		1										1				1				1			4
Preterm and hospitalized newborn health		1			1	1			1	1	1	1	3						1				11
Psychotic disorders	1	1	2						1			2	2									2	11
Stroke	2					1	1						1				1		1			1	8
Total	11	18	11	11	18	28	6	3	8	16	15	18	37	6	13	12	7	4	7	5	6	58	
Number of sets that include the outcome	8	18	10	10	17	21	6	3	7	14	12	16	25	5	6	9	7	4	7	4	6	25	

The numbers in each cell represent the number of outcomes covering the respective PRO concept included in the ICHOM Standard Set Reference Guide

In total, 176 PROMs (of which 114 are unique) are recommended for measuring the 307 PROs. The number of different PROMs recommended for the 22 unique PRO concepts varies from four (for measuring urinary symptoms) to 28 (for measuring physical function). Not many PROMs are included in more than one Standard Set: the PROMIS Global Health is included in twelve Standard Sets, the Patient Health Questionnaire (PHQ) in six Standard Sets, World Health Organization Disability Assessment Schedule (WHODAS-2) in six Standard Sets, EuroQol 5D (EQ-5D) in five Standard Sets, European Organization for Research and Treatment for Cancer Quality of Life Questionnaire (EORTC QLQ-C30) in four Standard Sets, Kidscreen in four Standards Sets, and another 22 PROMs are included in two or three Standard Sets.

**Fig. 1:**
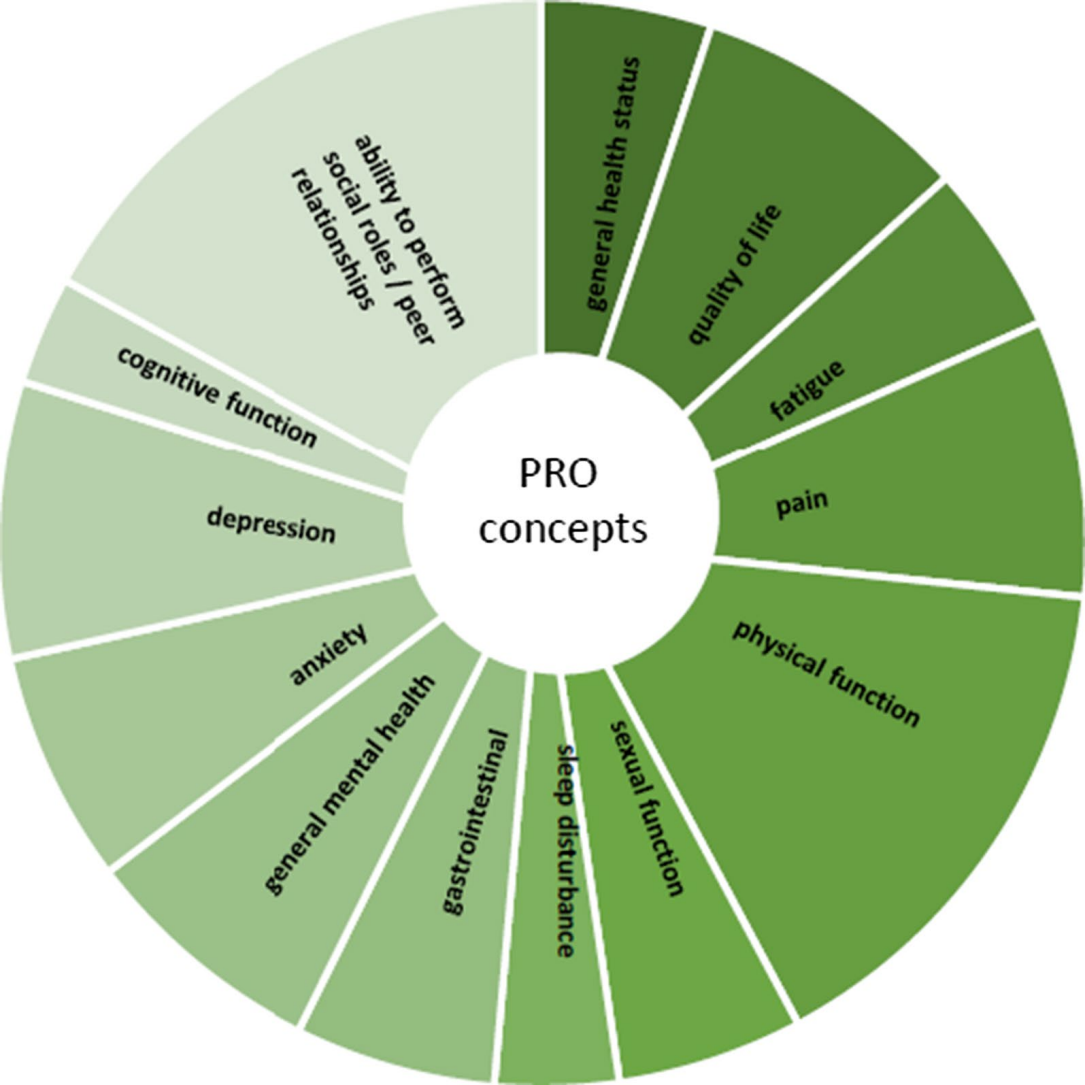
Most commonly included PROs included in ICHOM Standard Sets that can be measured with PROMIS (the size of each wedge represents the relative number of ICHOM sets (out of 39 sets) that include this PRO concept, the shading does not have a particular meaning)

More than half (17/22) of the PRO concepts (covering about 75% of the extracted PROs and also about 75% of the recommended PROMs) can be measured with PROMIS. Table 3 shows which PROMIS item banks are available to measure these PRO concepts. Figure 1 shows the most commonly included PROs in ICHOM Standard Sets that can be measured with PROMIS.

**Table 3:** Common PROs that can be measured with PROMIS

PRO	PROMIS adult item bank	Example item	PROMIS pediatric item bank	Example item
General health status (including general physical health and general mental health)/health-related quality of life	PROMIS v1.2 Global Health	In general, would you say your health is	PROMIS v1.0 Global Health	In general, would you say your health is
Quality of life	PROMIS Global02	In general, would you say your quality of life is		
Fatigue	PROMIS v1.0 Fatigue	How often did you feel tired?	PROMIS v2.0 Fatigue	I had trouble finishing things because I was too tired
Pain	PROMIS v1.1 Pain Interference	How much did pain interfere with your day to day activities?	PROMIS v2.0 Pain Interference	It was hard for me to pay attention when I had pain
	PROMIS v2.0 Pain Behavior	When I was in pain I lay down	PROMIS v1.0 Pain Behavior	I protected the part of my body that hurt
	PROMIS v1.0 Pain Intensity	How intense was your pain at its worst?	PROMIS v1.0 Pain Intensity	How bad was your pain on average?
	PROMIS v2.0 Pain Quality	Did your pain feel like pins and needles?	PROMIS v2.0 Pain Quality	Did your pain feel awful?
Physical function	PROMIS v2.0 Physical Function	Are you able to sit on and get up from the toilet?		
	PROMIS v2.0 Mobility	Are you able to go for a walk of at least 15 min?	PROMIS v2.0 Physical Function—Mobility	I could walk up stairs without holding on to anything
	PROMIS v2.0 Upper Extremity	Are you able to wash your back?	PROMIS v2.0 Physical Function—Upper Extremity	I could pull a shirt on over my head by myself
Sexual function	PROMIS v2.0 Sexual Function and Satisfaction	How satisfied have you been with your sexual relationship(s)?		
Sleep disturbance	PROMIS v1.0 Sleep Disturbances	I had difficulty falling asleep	PROMIS v1.0 Sleep Disturbances	I had difficulty falling asleep
Gastrointestinal symptoms	PROMIS v1.0 Gastrointestinal Symptoms	How often did you feel bloated?		
Anxiety	PROMIS v1.0 Anxiety	I felt anxious	PROMIS v2.0 Anxiety	I felt scared
Depression	PROMIS v1.0 Depression	I felt sad	PROMIS v2.0 Depressive symptoms	I felt sad
Cognitive function	PROMIS v2.0 Cognitive Function	I have had trouble concentrating	PROMIS v2.0 Cognitive Function	It is hard for me to pay attention to one thing for more than 5–10 min
	PROMIS v2.0 Cognitive Function Abilities subset	My memory has been as good as usual		
Ability to participate in social roles/peer relationships	PROMIS v2.0 Abilities to Participate in Social Roles and Activities	I have trouble doing all of the family activities that I want to do	PROMIS v2.0 Peer Relationships	I felt accepted by other kids my age
	PROMIS v2.0 Satisfaction with Social Roles and Activities	I am satisfied with my ability to do things for fun with others		

*PROMIS* Patient-Reported Outcomes Measurement Information System

## Discussion

We identified 307 different PROs in 39 ICHOM Standard Sets. The 307 PROs refer to 22 unique PRO concepts. A total of 114 different PROMs are being recommended for measuring these 307 PROs. Seventeen of the 22 unique PRO concepts, covering about 75% of the PROs and also about 75% of the PROMs, can be measured with PROMIS.

There is room for harmonization of PROs across ICHOM Standard Sets. While value-based healthcare is trying to eliminate the silos between medical specialties, it currently seems to have created new silos between conditions. Some PROs are included in only a few Standard Sets, while they seem relevant for many patients. For example, fatigue is included in the Standard Sets for inflammatory arthritis and heart failure, but not in the Standard Set for coronary artery disease. Sleep disturbances is only included in seven of the 39 Standard Sets, although it is a common symptom in many other diseases [45]. These results may partly be explained by a selection of only the most relevant PROs per condition. However, it is questionable to what extend this ranking represents the patient’s perspective. A more universal and standardized approach, focusing on a core set of PROs that are relevant for most patients, may provide more opportunities for patient-centered care. The profile domains from the PROMIS conceptual framework, including Fatigue, Pain Intensity, Pain Interference, Physical Function, Sleep Disturbance, Anxiety, Depression, and the Ability to Participate in Social Roles and Activities (included in the PROMIS-29 Profile measure [46] and also available as CATs), have been found relevant to many disease populations [47–52] and seem to be a good starting point. The core PROs could then be supplemented with disease-specific PROs (e.g. disease-specific symptoms) where needed, covering the additional 25% of PROs included in the ICHOM sets that cannot not be measured with PROMIS.

We also found large differences in PRO terminology used. For example, the PRO concept physical function is included in 21 Standard Sets, but only 10 Standard Sets use a similar term, i.e. ‘physical function(ing)’. Other terms used are ‘activities of daily living’, ‘activity limitations’, ‘disability’, ‘functional status’, ‘mobility’, and ‘motor function’. It is unclear whether these terms really refer to different concepts or whether they actually refer to the same concept. Definitions of the PROs are mostly lacking in the ICHOM reference guides. Eighteen Standard Sets included the PRO ‘health-related quality of life’. This is a very broad concept and can cover different PRO concepts. It is unclear whether this term refers to the same concept or to different concepts across Standard Sets. More attention need to be paid to the terms and definitions of PROs to be measured. ICHOM has recently started to harmonize terminology across Standard Sets, which may improve this situation in the near future.

Many different PROMs are being recommended, even when assessing the same PRO. For some patient groups a disease-specific PROM is recommended, while for another patient group, a domain-specific or generic PROM is recommended to measure the same PRO. For example, to measure depression, disease-specific instruments (e.g. the Movement Disorders Society Unified Parkinson Disease Rating Scale (MDS-UPDRS) for Parkinson), a cancer-specific instrument (EORTC QLQ-C30), several domain-specific instruments (e.g. Hospital Anxiety and Depression Scale (HADS), PHQ-2, PHQ-9, WHO-5) and several generic instruments (e.g. PROMIS-29 Profile, PROMIS Global Health and 36-Item Short Form Health Survey (SF-36)) are being recommended. Furthermore, nine different PROMs are being recommended to measure fatigue. This variability may partly be explained by (lack of) available evidence to support the use of a particular PROM in a specific condition. It is time-consuming and costly to translate and validate so many PROMs across countries. Recommending different PROMs for the same PROs hampers outcome measurement in daily clinical practice and comparisons across patient groups with different conditions. Harmonization of PROMs can improve this situation. For example, a PROMIS Depression or Fatigue measure could be used across all patient groups for which these PROs are relevant. Research has shown that it may not be necessary to measure common symptoms like fatigue or depression with a different instrument, validated for each different patient group. A study in patients with rheumatoid arthritis, for example, found that up to 90% of patients with arthritis would rate their level of fatigue similarly when asked in a general sense about fatigue or when asked about the fatigue they attributed to their rheumatoid arthritis, which suggest that a generic PROM can be used instead of a disease-specific PROM [53]. Furthermore, evidence is growing for the validity of generic PROMIS measures across patient populations [47–52, 54].

Another issue we identified is that there is often not an exact match in the ICHOM sets between the PROs and PROMs. This means that not every PRO is measured with a separate (sub)scale. For example, in the ICHOM Standard Set for Overall Adult Health, five PROs are included that address mental health (general mental health, sleep, depression, vitality, and anxiety). Two PROMs are recommended (PROMIS Global Health and WHO5) for measuring these PROs. However, neither the PROMIS Global Health nor the WHO5 provide separate scores for sleep, depression, vitality, and anxiety. If PROMs are to be used in the consultation room, separate scores for each PRO may be more helpful.

We argue that there is room for harmonization of PROs and PROMs across ICHOM Standard Sets. Our study showed that many PROs that matter to patients are common across patient groups. It may not be necessary to use a different PROM to measure common PROs, like pain, fatigue, depression, in different patient populations. It is hard to identify the best PROM for a specific patient population because the number of validation studies of PROMs in specific patient populations is limited and evidence on important measurement properties (e.g. responsiveness) is often lacking (See for example [55–57]). It is too expensive and time-consuming to develop, validate, translate, and maintain different high quality PROMs for every patient group. It is also too expensive, complex and time-consuming to implement all these different PROMs in electronic health records, give the right PROM to the right patient, interpret the scores for every PROM in the correct way, and discuss them appropriately with patients in the consultation room. It is burdensome and confusing for patients with multi- or comorbidity to complete multiple, partly overlapping, PROMs for every health care professional they consult.

To go forward, we recommend a more universal and standardized approach to PRO and PROM selection. Much can be gained by selecting common PROs and PROMs across conditions wherever possible, for example using the conceptual framework and measures of PROMIS. There are several initiatives ongoing in this direction. One is the recently developed ICHOM adult overall health and pediatric overall health Standard Sets [58, 59]. These Standard Set contain 15 and 10 PROs respectively, to be measured in all adult or pediatric patients. Eleven of the 15 PROs for adults and eight of the 10 PROs for children the can be measured with PROMIS. The relevance and feasibility of measuring a common set of PROs in all patients needs to be evaluated. In the Netherlands, an alternative approach is being proposed. A national PROM working group developed a ‘menu’ of commonly relevant PROs and recommended PROMs, that can be used to select relevant PROs for a specific patient group(s) [60]. Both the ICHOM overall health Standard Sets and the Dutch ‘menu’ recommend measuring generic PROs where possible, supplemented with disease-specific PROs where needed. An approach which may be referred to as 'generic unless'. Both initiatives include PROMIS measures in their recommendations to standardize and reduce the number of PROMs being used. PROMIS measures are also included in 14 out of the 39 ICHOM Standard Sets. There is, however, much room left for improving the efficiency and validity of ICHOM Standard Sets.

PROMIS has several advantages over traditional PROMs. Since it is applicable across disease populations, it enables benchmarking, learning and improving quality of care in patients groups with multimorbidity, the main cost-drivers of healthcare, and across many of these patient groups. Moreover, it is also suitable for patients without a definite diagnosis or for patients with rare diseases, for which validated disease-specific PROMs are quite often not available. An additional advantage of PROMIS is the possibility of CAT [37]. With CAT the computer selects items from an item bank, based on answers to previous items. This yield highly reliable scores with a few relevant items only, which is an important benefit for using PROMs in clinical practice. Technical solutions for CAT application are currently available in a limited number of countries, but this is expected to increase in the near future. As long as these technical solutions are lacking, PROMIS short forms can be applied as an alternative. Finally, PROMIS is a sustainable system, maintained by the PROMIS Health Organization, an international network of researchers and clinicians across a large number of countries, who collaborate to facilitate widespread use and adoption of PROMIS in research and clinical practice.

A limitation of our study is that the classification of PROs was not done by raters independently. The initial classification was done by one rater, and then reviewed with confirmation by a second rater. Furthermore, classification was based on information in the ICHOM reference guides only. We did not map the recommended PROMs on item level to the PROMIS measures. Our classification may therefore be not completely correct. However, the exact numbers are not important for our call for a more universal and standardized approach to PRO and PROM selection.

## Conclusion

We found considerable overlap in selected PROs across ICHOM Standard Sets, and large differences in terminology and recommended PROMs for the same PROs. For measuring 307 different PROs, covering 22 unique PRO concepts, a total of 114 different PROMs are currently being recommended. We recommend a more universal and standardized approach to the selection of PROs and PROMs. PROMIS offers an evidence-based conceptual framework of commonly relevant PROs and provides a sustainable set of validated PROMs, that are applicable across patient populations and medical specialties.

## Data Availability

An excel file with all data extraction is available upon request from the corresponding author.

## Abbreviations

CAT: Computerized Adaptive Test
EORTC QLQ-C30: European Organization for Research and Treatment for Cancer Quality of Life Questionnaire
EQ-5D: EuroQol 5D
HADS: Hospital Anxiety and Depression Scale
ICHOM: International Consortium for Health Outcomes Measurement
IRT: Item Response Theory
MDS-UPDRS: Movement Disorders Society Unified Parkinson Disease Rating Scale
PHQ: Patient Health Questionnaire (PHQ)
PRO: Patient-Reported Outcome
PROM: Patient-Reported Outcome Measure
PROMIS: Patient-Reported Outcomes Measurement Information System
SF-36: 36-Item Short Form Health Survey
WHODAS: World Health Organization Disability Assessment Schedule
